# Clinical research updates

**DOI:** 10.1111/camh.70131

**Published:** 2026-09-18

**Authors:** Marinos Kyriakopoulos, Fotios Cheimaras, Asimina Palaiologou, Vasiliki Kyriakopoulou

**Affiliations:** ^1^ National and Kapodistrian University of Athens Athens Greece; ^2^ Department of Child and Adolescent Psychiatry, Institute of Psychiatry Psychology and Neuroscience King‘s College London London UK

## Linking prolonged childhood and adolescent loneliness to schizophrenia spectrum disorders

Fotios Cheimaras

National and Kapodistrian University of Athens

Loneliness in childhood and adolescence has been linked to a range of adverse mental health outcomes, yet its role in the development of schizophrenia spectrum disorders (SSD) remains insufficiently studied. Although prior work has established a bidirectional relationship between loneliness and psychosis – with loneliness being more common in people with schizophrenia and worsening paranoia, hallucinations and depressive symptoms – little is known about the long‐term effects of prolonged early loneliness or how it interacts with genetic liability for schizophrenia, positioning loneliness as a form of subjective adversity, distinct from established adverse childhood experiences such as abuse and neglect.

Andreu‐Bernabeu et al. (2026) examined the association between prolonged childhood and adolescent loneliness (CAL) and SSD risk, drawing on the European Gene–Environment Interactions in Schizophrenia (EU‐GEI) case–control study. The study initially included 1261 patients with SSD (per DSM‐IV‐TR), 1282 unaffected siblings and 1525 healthy controls from Spain, Turkey and Serbia; after quality control and selection of participants with available genetic data and loneliness measures, the final analytic sample comprised 1037 patients, 1193 siblings and 1100 controls. Prolonged loneliness was assessed retrospectively with a single item (feeling lonely for ≥6 months) across two developmental periods: before age 12 and between ages 12–16. Genetic liability was captured with a schizophrenia polygenic risk score (PGS), dichotomised at the top quartile (>75%) of controls, while psychotic symptoms were measured with the CAPE‐42 scale. Gene–environment interactions were examined using mixed regression models and the Relative Excess Risk due to Interaction (RERI) method, with stratification by gender; predictive performance metrics (sensitivity, specificity and positive predictive value) were computed via Monte Carlo simulation, assuming a 1% prevalence of schizophrenia.

Prolonged loneliness was strongly associated with increased SSD risk, more so for adolescent than childhood loneliness (OR = 7.26 vs. 5.20), and remained significant after adjusting for current depressive symptoms. Weaker but comparable associations were seen in unaffected siblings, suggesting shared familial and environmental contributions. Both childhood and adolescent loneliness were linked to greater severity of positive, negative and depressive symptoms, with a gradient in effect from patients through siblings to controls. Critically, a significant additive interaction emerged between high genetic liability and loneliness on SSD risk (RERI = 23.46 for adolescent loneliness) – roughly four times that previously reported for cannabis use in the same cohort; the magnitude of the loneliness effect was comparable to that of established risk factors such as cannabis use or sexual abuse. This effect was more pronounced in women, both for the individual association (OR = 10.04 vs. 5.50 in men) and for the interaction with genetic liability. Importantly, the interaction was not attributable to gene–environment correlation, as no association between PGS and loneliness was found among controls. Adding the interaction term improved the discriminative ability of the predictive models (AUC from ~0.68 to ~0.76) and, more importantly, positive predictive value: from under 3% for genetic risk alone to 17% for individuals with high genetic liability and adolescent loneliness, reaching 22.5% in women and 26% in those with very high genetic liability (>90th percentile).

The study – the first to examine prolonged childhood and adolescent loneliness and its interaction with genetic liability in SSD risk – underscores that loneliness, particularly during adolescence, is a substantial environmental risk factor acting synergistically with genetic vulnerability. The authors note as key limitations the retrospective, single‐item assessment of loneliness and attendant recall bias, the use of a proxy measure for social isolation, the small subgroups after stratification by genetic risk and gender and limited generalisability owing to the predominantly European ancestry of the sample; they also note that predictive performance metrics were derived within the same sample. Given that loneliness is a potentially modifiable factor and can be identified in both clinical and community settings, its early detection could support risk stratification and the development of targeted preventive interventions, particularly in individuals with heightened genetic liability and especially in women.

Andreu‐Bernabeu, Á., González‐Peñas, J., Mora, A., Bernardo, M., Mezquida, G., Amoretti, S., … & Díaz‐Caneja, C.M. (2026). Linking prolonged childhood and adolescent loneliness to schizophrenia spectrum disorders: Results from EU‐GEI study. *The British Journal of Psychiatry*, 228(5), 428–436. https://doi.org/10.1192/bjp.2025.100

## Maternal and paternal antidepressant use before and during pregnancy and offspring risk of neurodevelopmental disorders

Asimina Palaiologou

National and Kapodistrian University of Athens

Antidepressant use before and during pregnancy is relatively common. Fathers may also receive antidepressant treatment before or around the time of conception. This has raised concerns about possible consequences for the child's neurodevelopment. Previous studies have reported associations between prenatal antidepressant exposure and neurodevelopmental conditions, particularly attention‐deficit/hyperactivity disorder (ADHD) and autism spectrum disorder (ASD). However, it has remained unclear whether these associations reflect an effect of the medication itself or the influence of parental mental health and shared familial, genetic or clinical factors.

To address this question, Chan and colleagues (2026) conducted a systematic review and meta‐analysis of the available evidence on neurodevelopmental outcomes following maternal or paternal antidepressant use before or during pregnancy. The analysis included 37 studies, covering 648,626 pregnancies exposed to antidepressants and approximately 25 million unexposed pregnancies.

The initial findings suggested a small association between antidepressant use during pregnancy and ADHD or ASD in offspring. No comparable association was identified for intellectual disability, motor disorders or speech and language disorders. Nevertheless, the certainty of the evidence was rated as low or very low. The associations with ADHD and ASD became weaker or were no longer statistically significant when the analyses accounted for potential confounding factors, including maternal mental health and shared genetic or familial influences. Importantly, studies comparing siblings from the same family found no increased risk of either ADHD or ASD. Antidepressant use by fathers during the pregnancy period served as a negative control and was also associated with both outcomes, despite the absence of direct foetal exposure to the paternal medication. Together, these observations suggest that the associations seen in the initial analyses may be explained to a considerable extent by shared genetic, environmental or family‐related factors rather than by medication exposure alone.

After more rigorous adjustment for confounding, no clear increase in risk was observed for individual selective serotonin reuptake inhibitors or serotonin–noradrenaline reuptake inhibitors. Possible exceptions were amitriptyline and nortriptyline, for which a potential safety signal was reported. However, this finding was based on limited evidence. These medications are also more likely to be prescribed in severe or clinically complex cases. It is therefore difficult to separate a possible medication effect from the influence of the severity and complexity of the underlying mental health condition. Further research is needed before firm conclusions can be drawn about these two medications.

The study has several limitations. There was substantial variation across the included studies, and some relevant factors could not be fully accounted for, including the severity of maternal depression, socio‐economic circumstances and the concurrent use of other medications. In addition, exposure was generally determined from prescription records rather than confirmed medication use. A prescription does not necessarily establish that the medication was taken or that foetal exposure occurred.

Overall, the findings suggest that antidepressants should not be stopped abruptly or avoided during pregnancy solely because of concerns about possible neurodevelopmental outcomes. Decisions require individualised care, careful assessment and clinical judgement, particularly because untreated maternal depression can also carry important risks for both the mother and the child.

Chan, J. K. N., Zhong, A. H. F., Lam, J. Y. H., Wong, C. S. M., Solmi, M., Correll, C. U., & Chang, W. C. (2026). Maternal and paternal antidepressant use before and during pregnancy and offspring risk of neurodevelopmental disorders: A systematic review and meta‐analysis. *The Lancet Psychiatry*, 13, 472–484. https://doi.org/10.1016/S2215‐0366(26)00089‐1

## The role of spirituality and religiosity in suicidal risk in adolescents and young adults

Vasiliki Kyriakopoulou

National and Kapodistrian University of Athens

Spirituality and religiosity seem to be, on the whole, protective factors in relation to suicidal thoughts and behaviours in adults. There have been several studies in adolescents supporting this too, but these had not been systematically reviewed.

West et al. (2025) conducted such a systematic review. They used PRISMA guidelines and searched PubMed, Embase, Web of Science and Cumulative Index to Nursing and Allied Health Literature (CINAHL) for articles published between 1 January 1998 and 18 December 2023. They included 61 studies of 340,170 adolescents and young adults aged 10–25 years. Studies were categorised as indicating protective associations if at least one protective and no risk associations between religiosity/spirituality and suicidality variables were reported, as indicating risk associations if at least one risk and no protective associations between religiosity/spirituality and suicidality variables were reported, and as indicating complex associations in mixed results. Studies were also evaluated through the Mixed Methods Appraisal Tool. The United States (24 studies) and the Middle East (8 studies) were overrepresented, and Africa (1 study) was underrepresented. In total, 67.2% of the studies showed protective associations, 8.2% risk associations, 8.2% complex associations and 16.4% no association. Due to the wide range of measures used, meta‐analysis was not conducted. Mediating variables included general social support, family support, degree of self‐esteem and substance use. Level of parental monitoring was identified as a moderating variable. Studies on ethnic minorities did not seem to indicate differences in results. In the few studies on religious and sexual and gender minorities, results were equivocal.

Limitations of the systematic review include the geographical distribution of the studies, with over‐ and underrepresentation of certain regions, and reporting on only certain religions. In addition, only observational and mostly cross‐sectional studies were identified and included, which do not allow determination of causality.

Overall, most studies indicated that religiosity and spirituality were associated with reduced suicidality in adolescents and young adults. Future research using experimental and longitudinal designs may help further clarify their role in suicidal risk reduction in youth. Collaboration of mental health services with religious institutions and spiritually informed care may assist in adolescent suicide prevention.

West, T., Rana, J., Awan, S., & Sagot, A.J. (2025). Systematic review: A 25‐year global publication analysis of the role of spirituality and religiosity in suicidal risk assessment in adolescents. *JAACAP Open*, 3(3), 347–378. https://doi.org/10.1016/j.jaacop.2025.01.003

## Conflict of interest statement

M.K. is the CAMH Associate Editor for Clinical Research Updates. The editor thanks the contributors for this issue's Clinical Research Updates. The editor has declared that he has no competing or potential conflicts of interest in relation to this article, and all remaining authors declare no competing or potential conflicts of interest.

## Funding information

The authors declare no funders for this article.

## AI statement

A.P. declares the use of AI in the following capacity: ChatGPT (OpenAI, GPT‐5) was used on 22 August 2026 for English‐language translation and linguistic editing. Specifically, AI assistance was used in the main text to improve grammar, clarity and readability. The author directed the process, reviewed all AI‐assisted revisions and takes full responsibility for the final content. F.C. declares the use of AI in the following capacity: ChatGPT (OpenAI, GPT‐5) and Claude (Anthropic, Claude Haiku 4.5) were used during July 2026 only for search purposes and grammar checking. AI was not used to generate any text. The text was written entirely by F.C., who takes full responsibility for its content and accuracy. V.K. declares that they did not use AI in any capacity that would require disclosure in accordance with Wiley's AI Guidelines, nor in any capacity that would reasonably require discloser for editors, reviewers and readers to properly evaluate their research or manuscript. All authors take full responsibility for the accuracy of this statement.

## Ethics statement

No ethical approval was required for these updates.

## Data Availability

Data sharing is not applicable to this article as no new data were created or analysed for this update.

